# Understanding Drug Release Data through Thermodynamic Analysis

**DOI:** 10.3390/ma10060651

**Published:** 2017-06-13

**Authors:** Marjorie Caroline Liberato Cavalcanti Freire, Francisco Alexandrino, Henrique Rodrigues Marcelino, Paulo Henrique de Souza Picciani, Kattya Gyselle de Holanda e Silva, Julieta Genre, Anselmo Gomes de Oliveira, Eryvaldo Sócrates Tabosa do Egito

**Affiliations:** 1 Faculdade de Farmácia, Universidade Federal do Rio Grande do Norte, Natal-RN 59012-570, Brazil; marjorie_freire_@hotmail.com; 2 Programa de Pós-graduaçãoem Nanotecnologia Farmacêutica, Universidade Federal do Rio Grande do Norte, Natal-RN 59012-570, Brazil; alexandrino_jr@yahoo.com.br; 3 Programa de Pós-graduaçãoem Ciências da Saúde, Universidade Federal do Rio Grande do Norte, Natal-RN 59012-570, Brazil; henrique.rmarcelino@gmail.com (H.R.M.); jgenre@gmail.com (J.G.); 4 Instituto de Macromoléculas Eloisa Mano, Universidade Federal do Rio de Janeiro, Rio de Janeiro-RJ 21941-598, Brazil; picciani@ima.ufrj.br; 5 Faculdade de Farmácia, Universidade Federal do Rio de Janeiro, Rio de Janeiro-RJ 21949-900, Brazil; holanda.gyselle@gmail.com; 6 Departamento de Fármacos e Medicamentos, Faculdade de Ciências Farmacêuticas, Universidade Estadual Paulista, Araraquara-SP 14800-903, Brazil; ans_gomes@yahoo.com.br

**Keywords:** Amphotericin B, drug release, kinetic profile, thermodynamics, hydrogels, films, nanofibers

## Abstract

Understanding the factors that can modify the drug release profile of a drug from a Drug-Delivery-System (DDS) is a mandatory step to determine the effectiveness of new therapies. The aim of this study was to assess the Amphotericin-B (AmB) kinetic release profiles from polymeric systems with different compositions and geometries and to correlate these profiles with the thermodynamic parameters through mathematical modeling. Film casting and electrospinning techniques were used to compare behavior of films and fibers, respectively. Release profiles from the DDSs were performed, and the mathematical modeling of the data was carried out. Activation energy, enthalpy, entropy and Gibbs free energy of the drug release process were determined. AmB release profiles showed that the relationship to overcome the enthalpic barrier was PVA-fiber > PVA-film > PLA-fiber > PLA-film. Drug release kinetics from the fibers and the films were better fitted on the Peppas–Sahlin and Higuchi models, respectively. The thermodynamic parameters corroborate these findings, revealing that the AmB release from the evaluated systems was an endothermic and non-spontaneous process. Thermodynamic parameters can be used to explain the drug kinetic release profiles. Such an approach is of utmost importance for DDS containing insoluble compounds, such as AmB, which is associated with an erratic bioavailability.

## 1. Introduction

The effectiveness of a pharmaceutical dosage form is usually dependent on the drug release and its dissolution in the biological fluids prior to its absorption. On the other hand, drug dissolution is a phenomenon that results from the combination of several factors [1], such as wettability and solubility of both the drug and excipients that compose the final product [2].

Molecules that are insoluble in water are usually associated with an erratic bioavailability, which is intrinsically related with the solubility and the permeability of the drug through the tissue [3]. Traditional attempts to solve this problem by generating an increment in the drug solubility by chemical and physical approaches have been successfully made [4]. The physical approaches are usually based on the incorporation of the insoluble drug into a drug carrier, which will be able to control the drug release and efficiently increase its bioavailability [5]. In this context, various drug carriers have been described in the last few decades [6], showing remarkable progress in the field of the pharmaceutical technology to increase therapeutics [7].

A wide variety of technologies has been used in order to modify the drug’s biopharmaceutical and pharmacokinetic properties, including liposomes, osmotic pumps, particulate systems, lipid carriers, polymeric systems, implants, enteric coatings, and others [8]. Among these, special attention has been paid to polymeric matrix systems, which are structures that control the release of active substances, dissolved or dispersed into the polymer matrix that can be resistant to disintegration [9].

The release of the drug from polymeric systems refers to the process by which drug molecules migrate from their initial position in the system to its outer surface [10]. This seemingly simple process is affected by many complex factors, such as the physical and chemical properties of the drug and the polymer, the structural characteristics of the system, the environment and the possible interactions among all of these factors [11].

The most common release mechanisms of drugs loaded in polymeric matrices are diffusion, erosion and swelling. The prevalence of a particular mechanism depends on the properties of the polymer used [12]. In general, drugs loaded into systems based on hydrophilic polymers are released from the matrix through water penetration into the system. This process can also be influenced by the temperature and the transition from a glassy to a matrix relaxation state [12]. Thus, in this type of matrix, the phenomena of swelling and diffusion are constantly found. However, for polymers of a hydrophobic nature, drug release occurs mainly by diffusion or erosion, one or the other prevailing according to the properties of the drug and the excipients employed [13].

Among several synthetic polymers found in the constitution of a drug delivery system, poly(vinyl alcohol) (PVA) and poly(lactic acid) (PLA) are largely described in the literature [14,15,16]. PVA is produced by the polymerization of vinyl acetate, presenting high solubility in water and high biocompatibility. Due to these characteristics, this polymer has been mainly used in topical and ophthalmic formulations, as well as a release control matrix. On the other hand, PLA is a hydrophobic, biodegradable and bioactive thermoplastic aliphatic polyester derived from renewable resources [17]. Like PVA, PLA is biocompatible and safe. Its degradation leads to the production of lactic acid, which is well tolerated by the body and does not induce significant immune responses [18]. In the course of the development of pharmaceutical formulations, PLA has been used in drug release systems, such as implants, injections and oral solid dispersions. It was also used as a coating agent for solid dosage forms [18].

The quantitative evaluation of the drug release from a delivery system is of great interest in the pharmaceutical field. Indeed, for a better understanding of this phenomenon, a wide variety of mathematical models has been developed to fit the experimental data [19]. These models represent release profiles obtained from the ratio between fractions of released drug versus time [20]. Among the most commonly-used models to describe release mechanisms are: (I) the zero order kinetics model, in which drug release happens on a linear basis [20]; (II) the first order kinetic model, which is a mathematical model not related to biological or physicochemical phenomena [20]; (III) the Higuchi model, which is linked to Fick’s first law and which is a model able to describe drug dissolution from a series of pharmaceutical drug dosage forms [21]; and (IV) the Korsmeyer–Peppas model, which is based on the release of a solute from a drug delivery system by other mechanisms. In this case, a couple of mechanisms, based on diffusion (Fickian transport) and relaxation of the polymeric chains due to the transition from a semi-rigid to a more flexible state (Transport Case II), prevail [22]. In an attempt to quantify the relative contributions of both phenomena, the Korsmeyer–Peppas model was incorporated into the (V) Peppas–Sahlin model by introducing constants that reflect the relative contribution of each mechanism, thus allowing the quantification of each parameter [23]. Finally, other models used to describe and explain drug release are (VI) the Hopfenberg model [24], which explains the release of erodible systems with various geometries containing drugs at their surface, and (VII) the Baker–Lonsdale model [25], which is based on the Higuchi model and explains the release of the drug contained in spherical matrices.

Knowledge about drug release mechanisms from polymeric systems showed significant improvement after the application of the mentioned mathematical models [2], which ranged from a simple phenomenological and empirical process to probabilistic and molecular explanations/reasoning. However, one of the key gaps that remains between practices and theory is the need to establish the thermodynamic parameters that describe and explain drug release from these devices. Despite the plethora of the literature regarding drug release from matrix systems, there is still no equivalent effort to correlate drug release profiles with their thermodynamic parameters [26]. Furthermore, the analysis of these parameters would provide a valuable tool to explain mechanisms by which the drug is released. Indeed, despite the free energy concept being a familiar tool in pharmaceutical sciences, thermodynamic analysis is not widely used as a means to understand drug release data [27].

The choice of a polymer and the type of device to be used as the drug delivery system depends on the release mechanism that is intended to be obtained, the route of administration to be used and the drug physicochemical characteristics. In this study, the drug of choice was Amphotericin B (AmB), an antibiotic whose molecule has an amphoteric and amphiphilic character [28]. Clinically recognized for its effectiveness against systemic fungal infections and leishmaniasis, the AmB molecule is highly water insoluble and poorly permeable through tissues. The physicochemical properties of AmB contribute to its low bioavailability, which explains the fact that the current dosage forms of this drug available on the market are restricted to intravenous administration [29]. Due to the therapeutic potential of AmB and the need for its administration by different routes to better reach the target tissues, a large number of studies that develop new delivery systems has been continually described [30].

In addition to the use of mathematical models of drug release, the activation energy (*E_a_*) of the process and other thermodynamic parameters such as enthalpy (Δ*H*), entropy (Δ*S*) and Gibbs free energy (Δ*G*) are important parameters that may help to explain the drug release from delivery systems. Therefore, the aim of this study was to assess the AmB kinetic release profiles from polymeric systems with different compositions (hydrophilic (PVA) or hydrophobic (PLA)) and geometries (planar (film) or cylindrical (fiber)) and to correlate these profiles with the aforementioned thermodynamic parameters.

## 2. Results and Discussion

### 2.1. Production of Systems

In this study, different polymeric systems were developed aiming to produce an AmB drug delivery system for topical administration. Since AmB is an amphiphilic drug with high molecular weight and peculiar physicochemical characteristics [31], polymers with different natures such as PVA (hydrophilic) and PLA (hydrophobic) were chosen to produce these drug delivery systems. Afterwards, systems with the same polymer composition and different geometries such as films and nanofibers were successfully produced.

**Figure 1:**
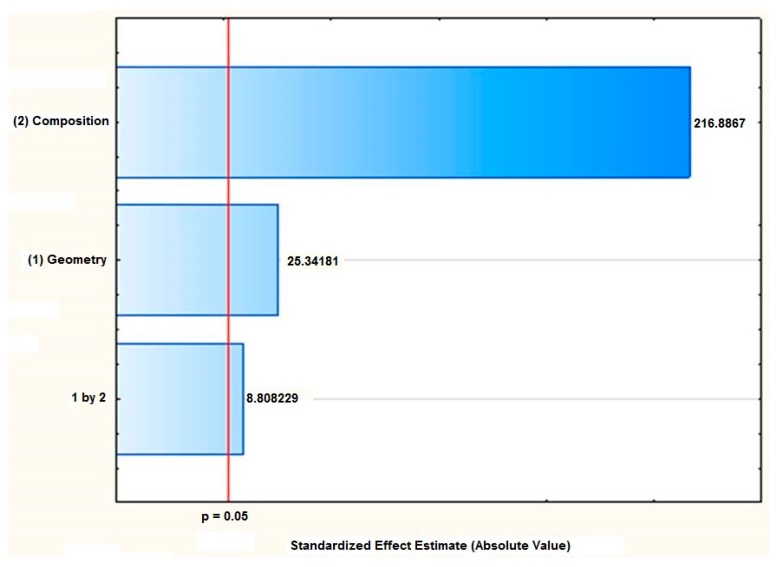
Pareto diagram demonstrating that the composition of the systems is a factor of higher magnitude affecting the percentage of the Amphotericin B released.

The statistical analysis of the influence of the polymer composition and the desired geometry showed that both factors were significant (*p* < 0.05), which was confirmed by the differences in their kinetic release profile. Therefore, an elaborated statistical model was obtained (Equation (A6)). The composition of the polymeric systems was the factor of the greatest effect magnitude over the amount of AmB released, as can be seen in the Pareto diagram (Figure 1). Indeed, the systems composed of PVA presented the highest percentage of drug release when compared to the PLA-based systems. This effect was attributed to the chemical nature of the polymers evaluated in which the hydrophilic polymer, PVA, provides a higher water penetration into the matrix, as can be seen by the wettability test, favoring the solvation of AmB contained therein and, consequently, the release process.

### 2.2. Scanning Electron Microscopy

**Figure 2:**
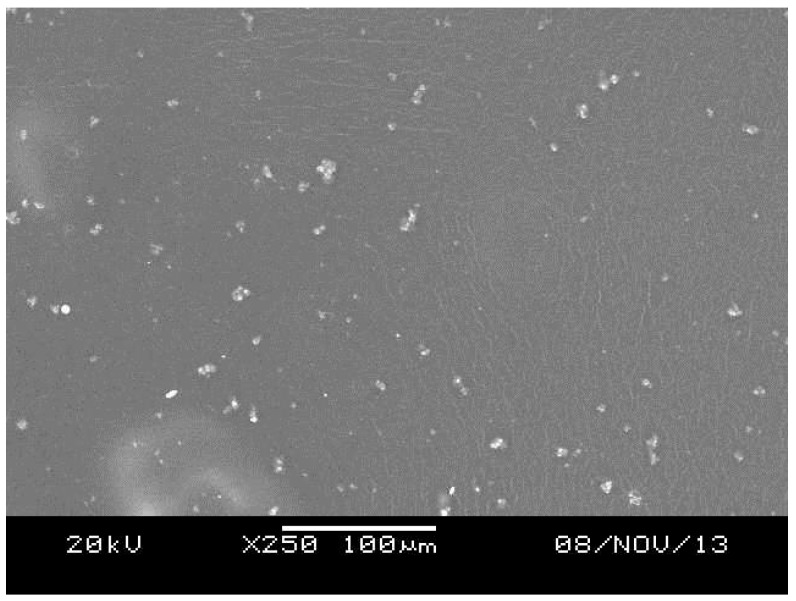
Scanning Electronic Microscopy (SEM) of the PVA film containing Amphotericin B.

**Figure 3:**
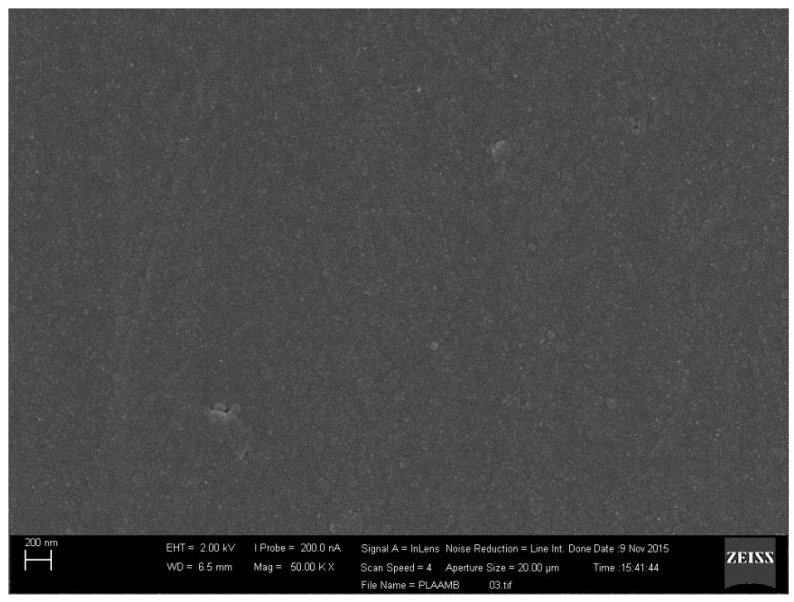
Scanning Electronic Microscopy (SEM) of the PLA film containing Amphotericin B.

**Figure 4:**
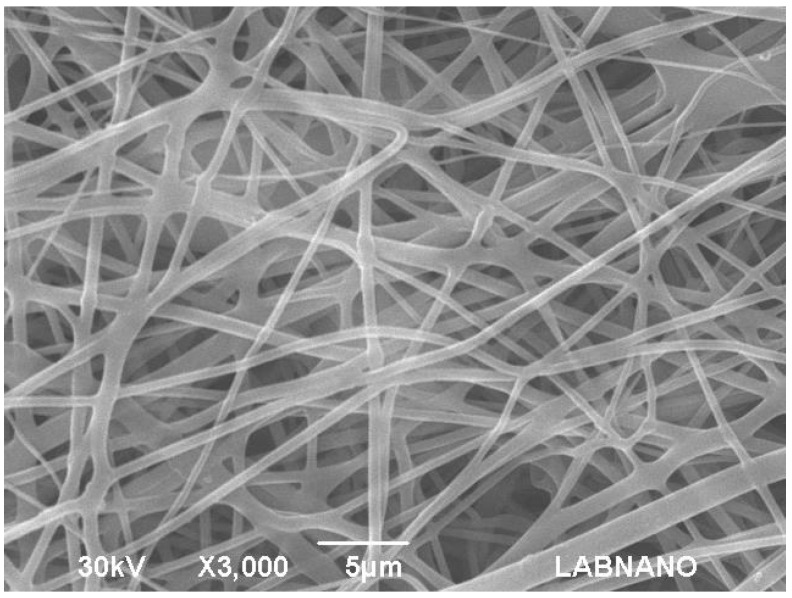
Scanning Electronic Microscopy (SEM) of the PVA fiber containing Amphotericin B.

**Figure 5:**
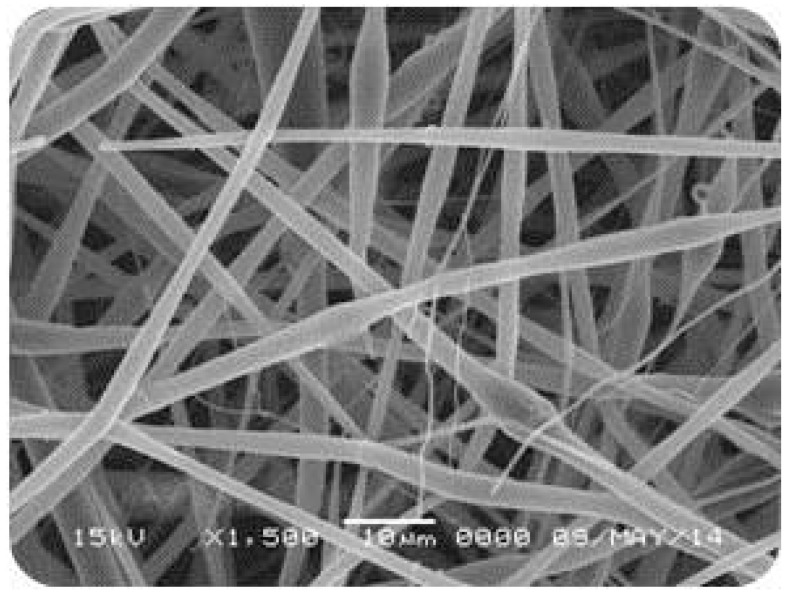
Scanning Electronic Microscopy (SEM) of the PLA fiber containing Amphotericin B.

The micrographic analysis of the PVA films revealed a smooth surface (Figure 2). Additionally, a slight precipitation of AmB that was not solubilized in the hydrophilic matrix was observed. On the other hand, the PLA films presented a homogeneous structure and no phase separation between the polymer and the drug (Figure 3). For the electrospinning systems, both fibers presented surfaces with pores and the average diameter of 350 ± 16 nm and 1.73 ± 10.4 µm for PVA (Figure 4) and PLA (Figure 5) fibers, respectively.

### 2.3. Superficial Wettability

**Figure 6:**
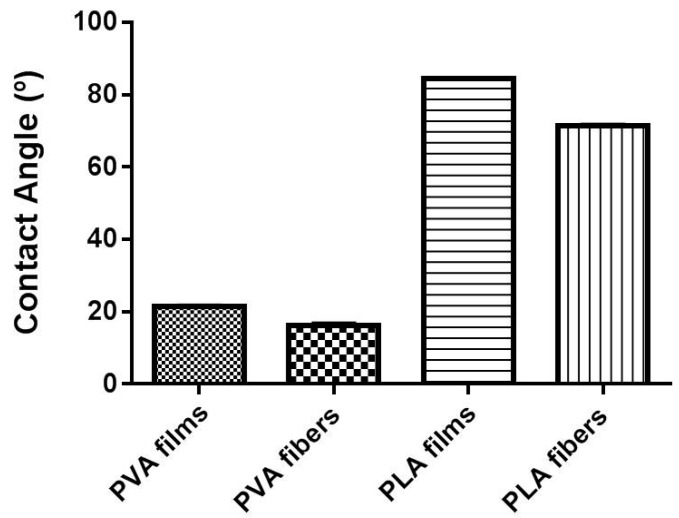
Superficial wettability of the evaluated systems.

Wettability is a parameter that describes the tendency of a liquid to wet (or spread over) a solid surface. This characteristic can be represented by the angle between the edge of the droplet surface and the liquid/solid interface, called the contact angle, which relates to the surface tension by Young’s equation [32]. Briefly, as the tendency of a drop to spread out over a solid surface increases, the contact angle decreases. Thus, the contact angle provides an inverse measure of wettability [33]. The measurements here performed showed that for the PVA samples, the wettability is higher when compared to those produced with the PLA, probably due to the inherent hydrophilicity of this polymer. Analysis of the resting angle between the wetting agent drop and the surface of the evaluated system indicated that a significant difference exists between the PLA systems (*p* < 0.05). Indeed, PLA-films, when compared to the same polymer fiber, had a higher contact angle indicating a lower wettability. On the other hand, by comparing the different PVA systems, fibers had a higher wettability than the film structure (*p* < 0.05) (Figure 6). Furthermore, it is comprehensible that electrospinning samples require greater energy to initiate the interaction between the medium and the polymer, probably due to their strong interactions among the polymer chains that form the fiber [31]. However, once this barrier was broken, the wettability was superior for PVA films.

### 2.4. Evaluation of the AmB Content in the Systems

PVA film had a concentration of 33.4 ± 0.1 µg AmB per milligram of dosed film, while the fiber of the same polymer showed 11.6 ± 0.2 µg of drug per milligram of fiber. For systems consisting of PLA polymer, film and fiber, these values were 8.6 ± 0.2 µg and 10.8 ± 0.2 µg of AmB by system milligrams, respectively (Table 1).

### 2.5. AmB Kinetics Release Profile

**Figure 7:**
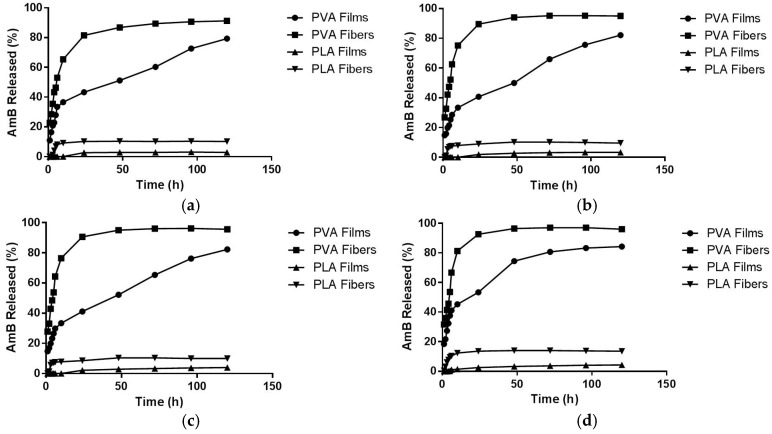
Kinetic release profiles of the systems at different temperatures: (**a**) 25 °C; (**b**) 32 °C; (**c**) 37 °C; (**d**) 45 °C.

The AmB release profiles obtained for all of the systems at different temperatures can be observed in Figure 7. A difference in the release profile was noticed between the hydrophilic evaluated systems. The PVA film had a greater controlled release, reaching its maximum percentage of drug released (84.3 ± 0.7%) after 120 h and a 20% burst release. On the other hand, PVA fibers presented a higher percentage of drug released with a higher burst release (around 59%), reaching the maximum percentage (97.05 ± 0.35%) after 72 h of the experiment. The behavior observed for PVA fibers could be due to the synergism between the absence of chemical cross linking and the increased surface area presented by this geometry, favoring the wetting process and, therefore, enhancing the AmB leakage.

Concerning the PLA systems, the drug release profile has also changed according to the system’s organizational structure. For PLA films, a low drug release profile was observed, probably due to the hydrophobicity of the polymer and its rigid structure, presenting a maximum AmB release of 4.12 ± 0.35% after 72 h. In the case of PLA fibers, similar to the PVA fibers, a better interaction with the solvent due to the greater wettability of the system was observed, explaining the greater drug release observed (14.02 ± 0.12% after 48 h) (Table 2). Regarding the different temperatures used for study, the variation between 25 °C and 45 °C had little effect on the drug release profiles (Table 2). In fact, as will be further explained, the drug release mechanisms in all systems studied here have an endothermic nature (Δ*H* > 0). Consequently, the differences seen when comparing the release profiles can be matched with the thermodynamic parameters calculated [34].

A possible explanation for the lower release profiles of the AmB from systems constituted by PLA when compared to those with PVA, in addition to the already mentioned hydrophobicity of PLA, could be a greater interaction of the drug with the polymer. It could be hypothesized that a possible interaction of AmB with PLA-chloroform may occur during the production of the system, leading to a greater drug-polymer interaction, thus hindering the further dissolution of AmB.

### 2.6. Mathematical Models of Releasing Kinetics

**Figure 8:**
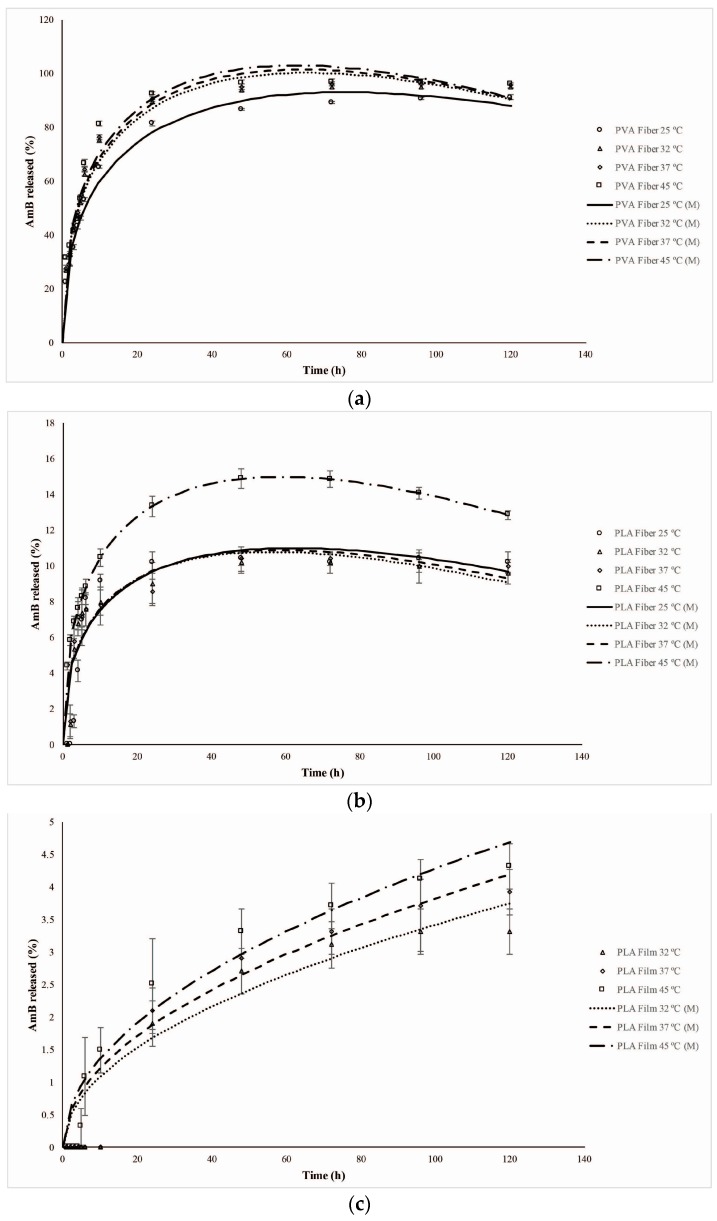

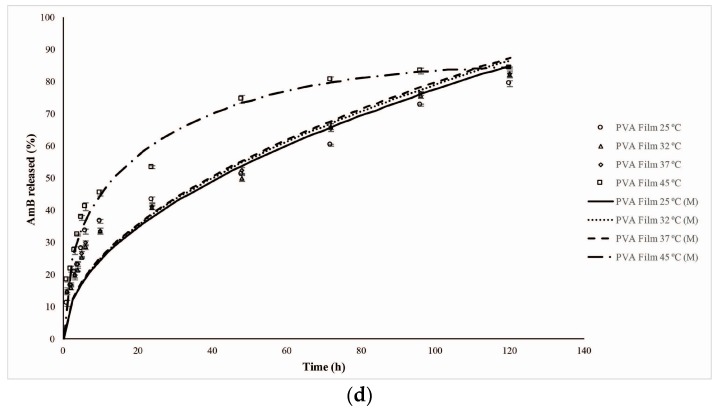
Adjustment of the experimental release data of the systems to the Peppas–Sahlin mathematical model at different temperatures. (**a**) PVA fibers; (**b**) PLA fibers; (**c**) PLA films; (**d**) PVA films. (M = Mathematical modeling).

The results obtained from the modeling (adjusted-*R*^2^ and RMSE) for each system evaluated at the specific temperatures, as well as their respective constant rates are shown in Table 3. The kinetic release profile presented by the electrospinning systems (PVA and PLA fibers) at all temperatures were better fitted by the Peppas–Sahlin model (Figure 8a,b), which explains that drug release occurs through coupling of the Fickian diffusion phenomena and the transition of the polymer from a semi-rigid to a flexible state, generating a relaxation of the polymer chains. The application of this model allows the estimation (Mathematical modeling (M)) of each of these mechanisms in the drug release process through the constants *k*_1_ and *k*_2_. Once *k*_1_ > *k*_2_, the Fickian diffusion is the major factor in drug release. However, when *k*_2_ > *k*_1_, the relaxation of the polymer chains is the predominant step [20]. The experimental data reveal predominance of the diffusion (positive values for *k*_1_) relative to the relaxation of the polymeric chains (Table 3).

For the PLA films, the release model with the best fit was the one proposed by Higuchi (Figure 8c). This model explains that the drug release process occurs only through Fickian diffusion and is commonly found in systems with hydrophobic constituents [20]. Since in this type of system, the water intake is limited and, therefore, the hydrolysis process significantly slow, the drug diffusion is the only mechanism to explain the AmB release from the PLA films in the studied conditions. For this system, the curve experimentally obtained at 25 °C did not provide a significant slope for its further mathematical modeling. Thus, the data modelling was not possible. However, due to the results obtained at the higher temperatures for the same system, it is possible to hypothesize that the AmB release may also occur by Fickian diffusion.

PVA films are hydrophilic polymeric structures in which the water is able to easily permeate, leading to the swelling of the matrix. At temperatures of 25, 32 and 37 °C, this system had the AmB release majorly determined by a Fickian diffusion process, which was best fitted by the Higuchi model. However, at 45 °C, the system showed a different dissolution profile, and the data were best fitted by the Peppas–Sahlin model (Figure 8d). The diffusion constant (*k*_1_) was higher than the chain relaxation constant (*k*_2_), which might mean that this increase in temperature caused a relaxation of the PVA chains (Transport Case II) that could have significantly influenced the drug release.

### 2.7. Thermodynamic Parameters

The analysis of the drug kinetics release at different temperatures proposed in this study allowed the application of a thermodynamic approach to its evaluation. Therefore, it was possible to determine the activation energy (*E_a_*) for the AmB release process in which high *E_a_* values indicated that a greater amount of energy was needed to move the drug from the polymer matrix to the dissolution medium [35].

Among the PVA-based systems, fibers had a higher *E_a_* when compared to films, 3.677 ± 0.074 kJ·mol^−1^ and 2.032 ± 0.066 kJ·mol^−1^, respectively. This is probably the result of the electrospinning process, which leads to the formation of an air surface layer over the porous structure (Figure 4), then requiring more energy for wetting to occur and to trigger the drug release process [36]. In contrast, among the PLA systems, films showed higher *E_a_*, equivalent to 12.908 ± 1.475 kJ·mol^−1^, which corroborates the lower wettability observed. The PLA fibers, despite the packaging promoted during the electrospinning [37], presented polymer unpacking in the dissolution medium, resulting in a small contact surface and, consequently, a smaller *E_a_* (1.763 ± 0.017 kJ·mol^−1^) to start the releasing process compared to the PLA films.

To calculate other thermodynamic parameters, the Eyring equation was used [38]. It is important to note that changes in the thermodynamic parameters also represent changes in the drug release behavior, which are, at least, under the influence of the surrounding environment, the type of polymer and the type of system. An enthalpy change between the four formulations may be the result of a change in the behavior of any of these component factors [39]. Additionally, the Δ*H* calculated suggests that all systems presented release processes of an endothermic nature (Δ*H* > 0) [34]. This assumption is in accordance with the results found in the experimental data, where there was an increase in the drug release when the temperature was higher, revealing that the release requires heat to occur. Furthermore, the systems’ entropy was negative (Δ*S* < 0), which characterizes a decrease in the system disorder, once the drug diffuses in the medium to try to reach an equilibrium between the amount inside and outside of the drug delivery [34].

The thermodynamic of the reaction process is correlated to the variations in the Δ*G*, the most important thermodynamic parameter associated with the release kinetics [40]. Furthermore, Δ*G* reveals the spontaneity of the process when a negative value is calculated for this parameter [41]. In this context, all systems had a positive free energy (Δ*G* > 0), which features a non-spontaneous natural process.

The thermodynamic data corroborate the findings of the release kinetic study, showing that systems that had higher percentages of drug release were those with the lowest free energy values. Indeed, the PLA film was the system that showed the greatest release limitation and, at the end, showed the highest values for the Gibbs free energy parameter. On the other hand, the PVA fibers had lower experimental Δ*G* values, indicating that the more free energy a process has, the greater the system's difficulty to release the drug from the polymeric matrix. The thermodynamic data obtained are summarized in Table 4, for all evaluated systems. For the PVA films, these parameters were obtained only for the first three evaluated temperatures in which the release mechanism was maintained. This was due to the fact that at 45 °C, the system changed its release behavior, and thus, it was not possible to determine the transition activation energy of the process.

**Figure 9:**
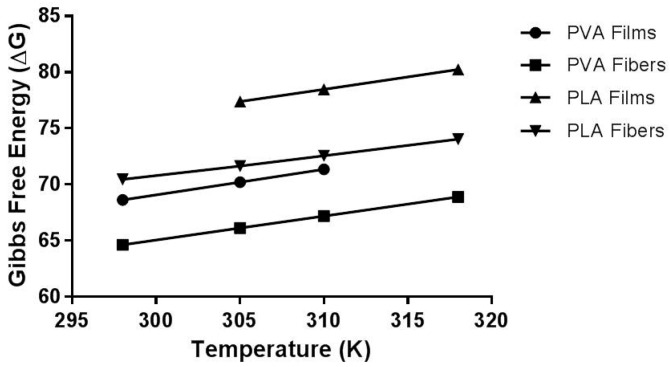
Relationship between the Gibbs free energy of the four systems evaluated as a function of the temperature.

The overall results show that the relationship to overcome the enthalpy barrier (*E_a_*) is PLA-film > PLA-fiber > PVA-film > PVA-fiber (Figure 9). From this study, it can be suggested that changes in the structural and the constitutive parameters of the pharmaceutical dosage forms induce synergistic changes for the system as a whole. Indeed, these changes are related not only to the drug release behavior, but also to the polymer nature, its molecular organization and the dissolution medium. It can be hypothesized that a change in the enthalpy between both formulations may be the result of a change in the behavior of any factor, concerning the composition and the geometry of the system. In this case, it was observed that a different interaction may exist between systems of similar composition that differ only in their geometrical organization.

## 3. Materials and Methods

### 3.1. Materials

Poly (vinyl alcohol) (98% hydrolysis degree, M_w_ 13,000–23,000 g/mol) and methanol came from Sigma-Aldrich^®^, St. Louis, MO, USA. Poly(lactic acid) (M_w_ 200,000 g/mol) was a donation from Natureworks, Minnetonka, MN, USA. Dimethylsulfoxide, glutaraldehyde (aqueous solution 25% (v/v)), sodium chloride (Pro Analyses), dibasic sodium phosphate (P.A.) and chloroform were purchased from Vetec^®^ Química Fina, Brazil. Potassium chloride (P.A.) was procured from Isofar, Brazil. Monobasic potassium phosphate (P.A.) was from Reagen, Brazil. Anforicin B (the amphotericin micellar system) was from Cristália (Itapira, São Paulo, Brazil), and amphotericin B drug powder (AmB) was purchased from Indofine Chemical Company, Inc. (Hillsborough, NJ, USA). 

### 3.2. Methods

#### 3.2.1. Development of Polymeric Systems

##### PVA Films

PVA films were prepared using the casting approach with a few modifications [42]. Briefly, PVA powder was accurately weighed, introduced in water and completely dissolved at 90 °C for 15 min to form a 10% (w/v) solution. The obtained solution was cooled slowly at room temperature under magnetic stirring. Moreover, the pH was adjusted to 2.0 with HCl 1M, and an amount of glutaraldehyde was added to obtain a concentration of 26 mM. Afterwards, the AmB was added to achieve a 10% (w/v) solution, followed by ultrasound bath for 10 min. The cross-linking reaction was carried out for 16 h in a polyethylene dish (diameter of 5.5 cm). The system was, then, dried at room temperature and protected from light.

##### PVA Fibers

The PVA fibers were prepared using the electrospinning technique [37]. Briefly, PVA powder was accurately weighed, introduced in water and completely dissolved at 90 °C for 15 min to achieve a 10% (w/v) solution. Afterwards, Fungizone^®^ was added to the PVA solution to achieve a 5% (w/v) solution. The electrospinning was carried out by means of a high voltage power supply (Model PS/FC60P02.0-11, Glassman High Voltage Inc., High Bridge, NJ, USA) and a digital controlled syringe pump (Model 100, KD, Scientific Inc., Holliston, MA, USA). Electrospinning parameters such as distance from the needle to the collection plate, voltage and flow rate were fixed at 12 cm, 17.5 kV and 0.5 mL/h, respectively.

##### PLA Films

Similar to the PVA film production, the PLA films were prepared using the casting approach. PLA was accurately weighed and dissolved in chloroform for 45 min by magnetic stirring to generate a 10% (w/v) solution. Afterwards, Fungizone^®^ was added to the PLA solution to form a 1.32% (w/v) solution, followed by ultrasound bath for 10 min. The solution was placed for 10 h in a glass dish (diameter of 5.5 cm). The films were dried at room temperature, protected from light.

##### PLA Fibers

As for the PVA fibers, PLA fibers were prepared using the electrospinning technique [37]. The PLA was accurately weighed and dissolved in chloroform for 45 min to produce a 15% (w/v) solution. Afterwards, Fungizone^®^ was added to the PLA solution to achieve a 1.32% (w/v) solution. The electrospinning was carried out by means of a high voltage power supply (Model PS/FC60P02.0-11, Glassman High Voltage Inc., NJ, USA) and a digital controlled syringe pump (Model 100, KD, Scientific Inc., Holliston, MA, USA). Electrospinning parameters such as distance from the needle to the collection plate, voltage and flow rate were fixed at 12 cm, 17.5 kV and 0.5 mL/h, respectively.

#### 3.2.2. Factorial Design and Statistical Analysis of the Systems

A 2^2^ full factorial design (Table 5) was used to evaluate the influence of two major factors: the polymeric composition of the matrix (PVA as a hydrophilic polymer and PLA as a hydrophobic one, respectively) and the geometry of the system (films with planar geometry and fibers with cylindrical geometry). Statistical analyses were performed using the GraphPad Prism 5.03 software (GraphPad^®^ Software, San Diego, CA, USA). Initially, the Shapiro–Wilk test was used to evaluate the normal distribution data, followed by the Bartlett test to evaluate the homogeneity of the variances. Finally, differences between the mean values were evaluated by applying the analysis of variance (ANOVA), and the differences between means were considered statistically significant when the *p*-value wasless than 0.05.

#### 3.2.3. Scanning Electron Microscopy 

SEM analyses were performed by JEOL JSM-5610 LV Scanning Electron Microscope (JEOL USA, Inc., Tokyo, Japan) to evaluate the morphology of the systems. The systems were placed on a carbon tape fixed on a stainless steel sample holder. Then, the samples were metalized with gold by the sputtering process. Analyses were performed using an acceleration voltage up to 30 kV. The electrospinning systems had their average diameter calculated by the Software 1.46r ImageJ (National Institutes of Health, Bethesda, MD, USA).

#### 3.2.4. Superficial Wettability

Surface wettability of the systems was determined by measuring the static contact angle of a water droplet using a goniometer coupled to a horizontal microscope (KSV CAM 200 Optical Tensiometer, KSV Instruments, Västra Frölunda, Sweden). Measurements were made at room temperature (22 °C) with relative air humidity varying from 40% to 50%.

#### 3.2.5. Drug Entrapment Efficiency

The amount of AmB entrapped into the system was measured with an analytical method previously validated. For drug extraction, 1 mg of each system was transferred to 3 mL of dimethyl sulfoxide (DMSO) and submitted to an ultrasound bath (USC-1800A-UNIQUE, UNIQUE, Indaiatuba, São Paulo, Brazil) for 15 min at room temperature. Moreover, the samples were analyzed by UV-Vis spectrophotometry (Libra S32 UV/Vis Spectrophotometer, Biochrom Ltd, Cambridge, UK) at the wavelength of 416 nm. Drug concentrations were calculated from the obtained absorbance value using an analytical curve of AmB in DMSO, previously developed (*y* = 97*x* + 0.01, *R*^2^ = 0.9998, “where *y* stands for the absorbance value, while *x* is AmB concentration”).

#### 3.2.6. Kinetic Release Assay

Once the amounts of drug loading of the systems were different (5 for PVA and 1.32 for PLA), the kinetics release assays were performed using fragments of the films or the fibers containing a total nominal amount of 125 µg of AmB, which represents 100% in the future calculations to produce the kinetics release profile. The systems were placed into a recipient with 25 mL of a Phosphate-Buffered Saline (PBS):methanol (80:20 v/v) mixture. These samples were incubated, under the temperatures of 25, 32, 37 and 45 °C and a tangential agitation at 100 rpm (TE-420-TECNAL, TECNAL, Piracicaba, São Paulo, Brazil). These temperatures cover a range of temperatures necessary for a good evaluation of the delivery system, the average of the human body temperature (from 32 to 37 °C), the room temperature (25 °C) and the temperature for predicting hyperthermia (45 °C) [5]. Aliquots were collected at specific times (1, 2, 3, 4, 5, 6, 10, 24, 48, 72, 96 and 120 h) and analyzed by UV-Vis spectrophotometry (Libra S32 UV/Vis Spectrophotometer, Biochrom Ltd, Cambridge, UK) at the wavelength of 409 nm. Drug concentrations were calculated from the absorbance value obtained using an analytical curve of AmB in PBS:methanol (80:20 v/v) previously developed (*y* = 0.033*x* + 0.0062, *R*^2^ = 0.9964).

#### 3.2.7. Mathematical Modeling of the Kinetics Release

The drug release data of the systems were modeled using the Excel^®^(Microsoft, Santa Rosa, CA, USA) Add-In-DDSolver [19], followed by the testing of the main mathematical models of dissolution (Table 6) along with the respective equations and parameters. The percentage of the drug released during the dissolution process was evaluated as a function of time and applied to mathematical models. The adjusted coefficient of determination (adjusted-*R*^2^) was used to compare the adjustment of the theoretical models to the experimental data. When comparing models with different numbers of parameters, the *R*^2^ tends to increase when the number of parameters increases [20]. The adjusted-*R*^2^ can be calculated from Equation (A1). The nearer to the unit this value is, the better the adjustment of the model to the data. Differently from the adjusted-*R*^2^, the square Root Mean Squared Error (RMSE) evaluates the difference between the values observed experimentally and those adjusted by the model, according to Equation (A2). The smaller the RMSE, the better the adjustment of the experimental data to the model. Thus, the model that better describes the experimental data will be the one that presents the biggest adjusted-*R*^2^ and the smaller RMSE.

#### 3.2.8. Thermodynamic Parameters

The performance of the release kinetics assays at different temperatures for all systems, in triplicate, allowed us to calculate the thermodynamic parameters. The activation energy (*E_a_*) was calculated by the Arrhenius equation (Equation (A3)), in which *E_a_* is the activation energy (kJ·mol^−1^), *K*_0_ is the Arrhenius constant, *k*_2_ is the reaction constant, *R_g_* is the universal gas constant (8314 J·mol^−1^·K^−1^) and T is the absolute temperature (Kelvin). The changes in the thermodynamic activation parameters, the Gibbs free energy (ΔG*), the enthalpy (ΔH*) and the entropy (ΔS*) were calculated using the Eyring equation (Equation (A4)) where K_b_ and h are the Boltzmann (Rg/N, 1.38 × 10^−23^ J mol^−1^ K^−1^) and Planck (6.62 × 10^−34^ J s^−1^) constants, respectively [19]. The relationship between the activation Gibbs free energy, the enthalpy and the entropy obeys Equation (A5) [43].

## 4. Conclusions

Different polymeric systems showed different AmB release profiles, with the PVA fiber system being the one with the highest percentage of drug released. The adjustment of the experimental data to the mathematical models showed that the spinning systems follow the Peppas–Sahlin model. Among the systems with film structure, both the PVA and the PLA fit the Higuchirelease model. However, PVA films underwent a transition mechanism in most of the evaluated temperatures, thus following the Peppas–Sahlin model. Regarding the thermodynamic data, the activation energy showed a close correlation to the systems’ wettability and the drug release kinetics. Other thermodynamic parameters such as enthalpy, entropy and free energy also agreed with and corroborated the kinetic data obtained for all evaluated systems. The ΔG were different for all studied systems, which shows that the AmB release was hindered, depending on the organization of the polymer chains and the hydrophobicity of the polymer used. The variation of unfavorable free energy was in line with the action of the sustained release of the product. Additionally, for a specific polymer, changes in the sample processing significantly altered the drug release rate; however, the mathematical model remained the same. Finally, this manuscript shows the utmost importance of the use of different temperatures on the drug release assays. Indeed, a short variation in this parameter can cause a change in the drug release kinetic profile. In in vivo situations, in which some of the temperatures used in this work might be present, the pharmacological response of the drug might be affected, as a slower or faster release would change the drug plasmatic concentrations.

## Figures and Tables

**Table 1:** Quantitative analysis of Amphotericin B (AmB) presented in the evaluated systems.

System	Concentration (µg of AmB/ mg of system)
PVA Films	33.4 ± 0.1
PVA Fibers	11.6 ± 0.2
PLA Films	8.6 ± 0.2
PLA Fibers	10.8 ± 0.2

**Table 2:** Time of the maximum percentage of the drug released as a function of the temperature.

System	Temperatures (°C/K)	% Release	Time (h)
PVA Films	25/298	79.27 ± 1.60	120
	32/305	82.10 ± 0.92	120
	37/310	82.30 ± 1.21	120
	45/318	84.32 ± 0.70	120
PVA Fibers	25/298	91.19 ± 0.92	96
	32/305	95.23 ± 0.61	72
	37/310	96.24 ± 1.05	72
	45/318	97.05 ± 0.35	72
PLA Films	25/298	3.11 ± 0.61	96
	32/305	3.31 ± 0.35	96
	37/310	3.52 ± 0.35	96
	45/318	4.12 ± 0.35	72
PLA Fibers	25/298	10.18 ± 0.61	48
	32/305	10.38 ± 0.35	48
	37/310	10.38 ± 0.31	48
	45/318	14.02 ± 0.12	48

**Table 3:** Adjustment of the mathematical model to the kinetic data and the in vitro release rate constants of the process according to the temperature variation.

SYSTEM	Temperature (°C/K)	Mathematical Model	Equation	*R*^2^ Adjusted	RMSE	Constants
PVA Films	25/298	Higuchi	$F=\;K_{H}\times t^{0.5}$	0.91	6.34	$K_{H}$ = 7.19 (min) ^−0.5^
	32/305			0.93	6.10	$K_{H}$ = 7.38 (min) ^−0.5^
	37/310			0.92	6.53	$K_{H}$ = 7.52 (min) ^−0.5^
	45/318	Peppas–Sahlin	$F=\;k_{1\;}\times t^{m}+\;k_{2\;}\times t^{2m}$	0.98	3.13	$k_{1}$ =18.85$/k_{2}$ = −1.05
PVA Fibers	25/298	Peppas–Sahlin	$F=\;k_{1\;}\times t^{m}+\;k_{2}\times t^{2m}$	0.98	3.65	$k_{1}$ =29.21$/k_{2}$ = −1.86
	32/305			0.97	4.70	$k_{1}$ = 30.13$/k_{2}$ = −2.33
	37/310			0.97	4.91	$k_{1}$ = 31.01$/k_{2}$ = −2.40
	45/318			0.95	6.05	$k_{1}$ = 32.08$/k_{2}$ = −2.50
PLA Films	25/298	–	–	–	–	–
	32/305	Higuchi	$F=\;K_{H}\times t^{0.5}$	0.87	0.56	$K_{H}$ = 0.34 (min) ^−0.5^
	37/310			0.85	0.67	$K_{H}$ = 0.38 (min) ^−0.5^
	45/318			0.90	0.55	$K_{H}$ = 0.43 (min) ^−0.5^
PLA Fibers	25/298	Peppas–Sahlin	$F=\;k_{1\;}\times t^{m}+\;k_{2\;}\times t^{2m}$	0.72	2.40	$k_{1}$ = 3.05$/k_{2}$ = −0.267
	32/305			0.73	1.77	$k_{1}$ = 3.58$/k_{2}$ = −0.279
	37/310			0.75	1.78	$k_{1}$ = 4.03$/k_{2}$ = −0.28
	45/318			0.82	1.95	$k_{1}$ = 4.79$/k_{2}$ = −0.38

**Table 4:** Activation energy and thermodynamic parameters of the Amphotericin B release process.

System	Activation Energy (*E_a_*, kJ·mol^−1^)	Enthalpy (Δ*H*, kJ·mol^−1^)	Entropy (Δ*S*, J/(kg·K))	Gibbs Free Energy (Δ*G*)	
PVA Films	2.032 ± 0.066	0.600 ± 0.015	−0.2282 ± 0.001	**Temperature (K)**	**Δ*G* (kJ·mol^−1^)**
				298	68.609 ± 0.455
				305	70.207 ± 0.467
				310	71.349 ± 0.474
				318	-
PVA Fibers	3.677 ± 0.074	1.162 ± 0.036	−0.2130 ± 0.001	**Temperature (K)**	**Δ*G* (kJ·mol^−1^)**
				298	64.612 ± 0.027
				305	66.106 ± 0.028
				310	67.171 ± 0.028
				318	68.877 ± 0.028
PLA Films	12.908 ± 1.475	10.961 ± 0.170	−0.2177 ± 0.001	**Temperature (K)**	**Δ*G* (kJ·mol^−1^)**
				298	-
				305	77.344 ± 0.344
				310	78.466 ± 0.350
				318	80.241 ± 0.332
PLA Fibers	1.763 ± 0.017	15.214 ± 0.284	−0.1849 ± 0.001	**Temperature (K)**	**Δ*G* (kJ·mol^−1^)**
				298	70.440 ± 0.547
				305	71.635 ± 0.412
				310	72.560 ± 0.414
				318	74. 040 ± 0.418

**Table 5:** Factorial design used on the production of the polymeric systems.

Factor	Level (−1)	Level (+1)
Geometry	Film	Fiber
Composition	PLA	PVA

**Table 6:** Mathematical models of the kinetics release.

Dissolution Mathematical Model	Equation	Parameter (s)
Zero Order	$F=\;k_{0\;}\times t$	$k_{0\;}$ ^(1)^
First Order	$F=100\times \left(1-\;e^{-k_{1}\times t}\right)$	$k_{1}$ ^(2)^
Higuchi	$F=\;K_{H\;}\times {\;t}^{0.5}$	$K_{H}$ ^(3)^
Korsmeyer–Peppas	$F=\;k_{KP}\times t^{n}$	$k_{KP}$^(4)^, n^(5)^
Hopfenberg	$F=100\times \left[1-\left(k_{HB}\times t\right)n\right]$.	$k_{HB}$^(6)^, n^(7)^
Baker–Lonsdale	$2/3\;\times [1-{\left(1-\;F/100\right)}^{2/3}]-\;F/100=\;k_{BL\;}\times t$	$k_{BL\;}$ ^(8)^
Peppas–Sahlin	$F=\;k_{1\;}\times t^{m}+\;k_{2\;}\times t^{2m}$	$k_{1\;}$ ^(9)^, $k_{2}$^(10)^, m^(11)^

^(1)^ The zero-order release constant; ^(2)^ the first-order release constant; ^(3)^ the Higuchi release constant; ^(4)^ the release constant incorporating structural and geometric characteristics of the drug-dosage form; ^(5)^ the diffusional exponent indicating the drug-release mechanism; ^(6)^ the combined constant in the Hopfenberg model $k_{HB}=\;k_{0}/(C_{0\;}\times \;a_{0})$, where $k_{0}$ is the erosion rate constant; $C_{0\;}$= the initial concentration of the drug in the matrix; and $a_{0}$= the initial radius for a sphere or cylinder structure or the half thickness for a slab; ^(7)^ *n* = 1, 2 and 3 for a slab, cylinder and sphere structure, respectively; ^(8)^ the combined constant in the Bakerd sphere structure $k_{BL}=\left[3\;\times D\times \;C_{s}/(r_{0}^{2}\;\times \;C_{0}\right)]$, where D is the diffusion coefficient, $C_{s}$ is the saturation solubility, $r_{0}$ is the initial radius for a sphere or cylinder structure or the half-thickness for a slab and $C_{0\;}$is the initial drug loading in the matrix; ^(9)^ the constant related to the Fickian kinetics; ^(10)^ the constant related to Case II relaxation kinetics; ^(11)^ the diffusional exponent.

## Appendix A

### Equation List

Equation (A1): Determination of the adjusted determination coefficient (*R*^2^ adjusted), where $n_{d}$ is the number of analyzed data and p the number of parameters.

(A1) $${R^{2}}_{adjusted}=1-\left(11R^{2}\right)\times \;\frac{\left(n_{d}-1\right)}{\left(n_{d}-p\right)}$$

Equation (A2): Determination of the mean square error root, where $y_{exp}$ and $y_{calc}$, respectively, are the value obtained experimentally and the value calculated by the model, and $n_{d}$ is the number of analyzed data.

(A2) $$\sqrt{MSE}=\;\sqrt{\frac{{\sum (y_{exp}-\;y_{calc})}^{2}}{n_{d}}}$$

Equation (A3): Arrhenius equation, where $E_{a}$ is the activation energy (kJ·mol^−1^), $R_{g}$ is the universal gas constant (8314 J/mol·K), T is the temperature (Kelvin), $K_{0}$ is the Arrhenius constant and $K_{2}$ is the constant of the reaction.

(A3) $$lnK_{2}=lnK_{0}-\;\frac{E_{a}}{R_{g}}\;\times \;\frac{1}{T}$$

Equation (A4): Eyring Equation, where $K_{b}$ is the Boltzma constant, h is the Plank constant, $R_{g}$ is the universal gas constant, $K_{2}$ is the reaction constant and T is the temperature (Kelvin).

(A4) $$\ln\left(\frac{K_{2}}{T}\right)=\;\left[\ln\left(\frac{K_{b}}{h}\right)+\;\frac{∆S}{R_{g}}\right]-\;\frac{∆H}{R_{g}}\times \;\frac{1}{T}$$

Equation (A5): Gibbs free energy equation, which relates the enthalpy (∆H), entropy (∆S) and temperature (T).

(A5) ∆G= ∆H− T×∆S

Equation (A6): Statistical model designed for the release of Amphotericin B (AmB) correlating both factors (A) geometry and (B) system composition.

(A6) AmB released=47.51+4.8A+41.06B+1.67AB
